# lincRNA-Cox2 regulates NLRP3 inflammasome and autophagy mediated neuroinflammation

**DOI:** 10.1038/s41418-018-0105-8

**Published:** 2018-04-17

**Authors:** Zhenyi Xue, Zimu Zhang, Hongkun Liu, Wen Li, Xiangdong Guo, Zhihui Zhang, Ying Liu, Long Jia, Yan Li, Yinghui Ren, Hongwei Yang, Lijuan Zhang, Qi Zhang, Yurong Da, Junwei Hao, Zhi Yao, Rongxin Zhang

**Affiliations:** 1 Laboratory of Immunology and Inflammation, Department of Immunology and Research Center of Basic Medical Sciences, Key Laboratory of Immune Microenvironment and Diseases of Educational Ministry of China, Tianjin Key Laboratory of Cellular and Molecular Immunology, Tianjin Medical University, Tianjin, 300070 China; 2 Department of Neurology and Tianjin Neurological Institute, Tianjin Medical University General Hospital, Tianjin, China; 3 Guangdong Province Key Laboratory for Biotechnology Drug Candidates, Guangdong Pharmaceutical University, Guangzhou, China

**Keywords:** Inflammasome, Inflammasome

## Abstract

Inflammasome activation plays key roles in host defense, but also contributes to the pathogenesis of auto-inflammatory, and neurodegenerative diseases. As autophagy is connected with both the innate and adaptive immune systems, autophagic dysfunction is also closely related to inflammation, infection, and neurodegeneration. Here we identify that lincRNA-Cox2, previously known as a mediator of both the activation and repression of immune genes expression in innate immune cells, could bind NF-κB p65 and promote its nuclear translocation and transcription, modulating the expression of inflammasome sensor NLRP3 and adaptor ASC. Knockdown of lincRNA-Cox2 inhibited the inflammasome activation and prevented the lincRNA-Cox2-triggered caspase-1 activation, leading to decreased IL-1β secretion and weakened TIR-domain-containing adapter-inducing interferon-β (TRIF) cleavage, thereby enhancing TRIF-mediated autophagy. Elucidation of the link between lincRNA-Cox2 and the inflammasome-autophagy crosstalk in macrophage and microglia reveals a role for lncRNAs in activation of NLRP3 inflammasome and autophagy, and provides new opportunities for therapeutic intervention in neuroinflammation-dependent diseases.

## Introduction

The NLR family protein NLRP3 is an intracellular signaling molecule that senses many pathogen-derived, environmental, and host-derived factors [1]. Secretion of proinflammatory IL-1β and IL-18 requires the activation of inflammasome, a multiprotein oligomer assembled to catalyze the splicing of the IL-1β and IL-18 into mature forms [2]. Inflammasome activation plays key roles in host defense, but also contributes to the pathogenesis of cancer, metabolic, auto-inflammatory, and neurodegenerative diseases [3]. Microglia are different from bone marrow-derived monocytes and macrophages [4]. In pathological conditions, microglia release pro-inflammatory cytokines and cytotoxic factors, this aggravates the progression of neurodegenerative diseases [5].

Autophagy is a fundamental eukaryotic pathway that has multiple effects on immunity. Autophagy is induced by pattern recognition receptors and, through autophagic adaptors, it provides a mechanism for the elimination of intracellular microorganisms. Autophagy has multitiered immunological functions that influence infection, inflammation and immunity [6]. In its classical form, autophagy is an important homeostatic process wherein cytoplasmic components are degraded in a double-membrane-bound autophagosome in response to stress. As autophagy is connected with both the innate and adaptive immune systems, autophagic dysfunction is closely related to inflammation, infection, neurodegeneration and cancer [7].

Long noncoding RNAs (lncRNAs), a recently discovered class of noncoding RNA, are longer than 200 bp and do not encode protein but are important for development, differentiation and metabolism [8, 9]. Many lncRNAs in mammals have been well-characterized to be involved in diverse biological roles, such as X-chromosome inactivation (*Xist* [10], *Tsix* [11]), imprinting (*H19* [12], *Air* [13]), trans-acting gene regulation (*HOTAIR* [14]) and the regulation of nuclear import (*Nron* [15]). In the field of immunology, emerging evidence has shown that lncRNAs play a key role in regulating immune functions and autoimmunity [16]. Recent studies suggested that the expression of lncRNAs varies widely during activation of the innate and adaptive immune response, among which lincRNA-Cox2, located at 51 kb upstream of the protein-coding gene *Cox2* (also known as *Ptgs2*), increased its expression by nearly 1000-fold after Tlr4 stimulation in CD11c^+^ bone marrow-derived dendritic cells [17]. LincRNA-Cox2 expression is also induced by Tlr2 ligands in a MyD88- and NF-κB-dependent manner in bone marrow-derived macrophages (BMDMs) [18]. Furthermore, lncRNAs have been revealed to be able to modulate important processes of immunity, such as the production of inflammatory mediators, cell differentiation and migration by regulating protein-protein interactions or by base pairing with RNA and DNA [19]. However, how the activation of NLRP3 inflammasome and autophagy could be regulated by lncRNA during immune responses remains largely unknown.

In this study, we examined the role of lincRNA-Cox2 in regulating autophagy in activated BV2 cells, BMDMs and primary microglia cells. Our work revealed that lincRNA-Cox2 can bind NF-κB p65 and promote its nuclear translocation and transcription, modulating *Nlrp3* and *Asc* expression. LincRNA-Cox2 knockdown inhibits NLRP3 inflammasome activation by reducing expression of the inflammasome *Nlrp3* and *Asc* after LPS stimulation. Decreased NLRP3 inflammasome activation suppressed caspase-1 activation and reduced the cleavage of TIR-domain-containing adapter-inducing interferon-β (TRIF), thereby derepressing TRIF-mediated ATG5-dependent autophagy. Subsequently, the enhanced autophagy limited the inflammasome activity, serving as a collaborative partner that augmented the inhibition of inflammasome-mediated inflammation in macrophages and microglia. Finally, in mouse model of microglia-mediated central nervous system (CNS) inflammation [4], lincRNA-Cox2 knockdown results in protection from experimental autoimmune encephalomyelitis (EAE), and exhibits notable increased resting microglia (CD11b^+^CD45^med^), inhibited IL-1β secretion and enhanced autophagy in CNS. Here our data improve the understanding of crosstalk between the inflammasome and autophagy, and reveal a role for lncRNAs in activation of NLRP3 inflammasome and autophagy, and provides new opportunities for therapeutic intervention in neuroinflammation-dependent diseases.

## Results

### LincRNA-Cox2 regulates ATG5-dependent autophagy in BMDM and microglia cells

**Fig. 1:**
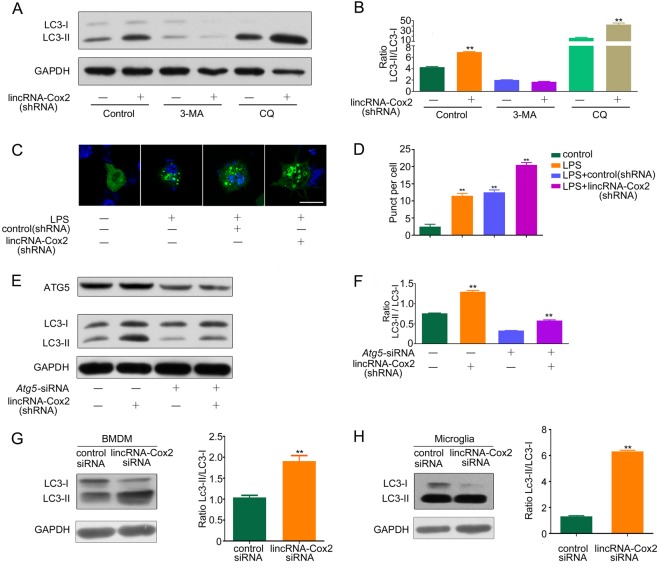
LincRNA-Cox2 regulates autophagy in an ATG5-dependent manner. **a** Western blot of LC3-I and LC3-II in control or lincRNA-Cox2 knockdown BV2 cells treated with LPS for 4 h and 1 mM ATP for 30 min, further treated in the presence or absence of 3-MA and CQ. **b** Ratio of LC3-II/LC3-I in three independent experiments from (**a**). **c** Representative immunofluorescence images of LC3 in control or lincRNA-Cox2 knockdown BV2 cells treated with LPS for 4 h and 1 mM ATP for 30 min. LC3 staining is shown in green, and nuclei are blue. Scale bar indicates 10 μm (three independent experiments). **d** Number of LC3 puncta in control or lincRNA-Cox2 knockdown BV2 cells treated with LPS for 4 h and 1 mM ATP for 30 min. **e** Effects of *Atg5* knockdown on LC3 II levels in control or lincRNA-Cox2 knockdown BV2 cells treated with LPS for 4 h and 1 mM ATP for 30 min. **f** Ratio of LC3-II/LC3-I in three independent experiments as in (**e**). **g**, **h** Western blot and the ratio of LC3-I and LC3-II in control or lincRNA-Cox2 knockdown BMDM and primary microglia cells treated with LPS for 4 h and 1 mM ATP for 30 min. GAPDH is shown as the loading control in all western blot figures (western blot repeated in three independent experiments). Columns are the mean values of triplicates; the error bar indicates the SEM. The symbol ** indicates a statistically significant difference from the control; *p* < 0.01

Autophagy can be triggered by Toll-like receptors [20–22]. To investigate the role of lincRNA-Cox2 in autophagy regulation and the mechanism through which lincRNA-Cox2 regulates autophagy, we used immunoblotting to detect LC3 levels in BV2 microglia cell lines stimulated by LPS. First, we confirmed the downregulated expression of lincRNA-Cox2 in shRNA-treated BV2 cells using qPCR and siRNA-treated BMDM using FISH (Supplementary Fig. 1). We also constructed 2 gRNAs targeting both 5’ and 3’ sequences flanking the lincRNACox2 locus and isolated a clone containing a deletion in the locus followed by verifying the loss of lincRNA-Cox2 expression by qPCR, serving as the lincRNA-Cox2 KO cell lines (Supplementary Fig. 1A). LincRNA-Cox2 knockdown by shRNA induced a marked increase in the conversion of LC3-I to LC3-II, which was increased by chloroquine diphosphate (CQ) and decreased by 3-methyladenine (3-MA) treatments (Fig. 1a). To quantify these results, the LC3-II/LC3-I ratio was analyzed and showed a marked increase when lincRNA-Cox2 was knocked down (Fig. 1b). To further confirm this result, we constructed the CRISPR/Cas9-induced lincRNA-Cox2-knockout cell lines and confirmed that knockout of lincRNA-Cox2 promoted the conversion of LC3-I to LC3-II (Supplementary Fig. 2). Also, a BV2 cell line stably expressing the autophagy marker GFP-LC3 was used to investigate LC3-II localization to autophagosomal membranes during autophagy using confocal microscopy, and lincRNA-Cox2 knockdown induced GFP-LC3 dot formation (Fig. 1c, d). Next, we tested the requirement of the *Atg5* gene for autophagy, and cells without Atg5 showed a marked reduction in autophagy (Fig. 1e, f). We also confirmed the enhanced autophagy in BMDM and primary microglia cells of lincRNA-Cox2 knockdown. The western blot and quantified results showed that lincRNA-Cox2 knockdown induced a marked increase in LC3-II/LC3-I ratio (Fig. 1g, h).

### LincRNA-Cox2 knockdown upregulates autophagy by inhibiting expression of inflammasome sensor Nlrp3 and the adaptor Asc

**Fig. 2:**
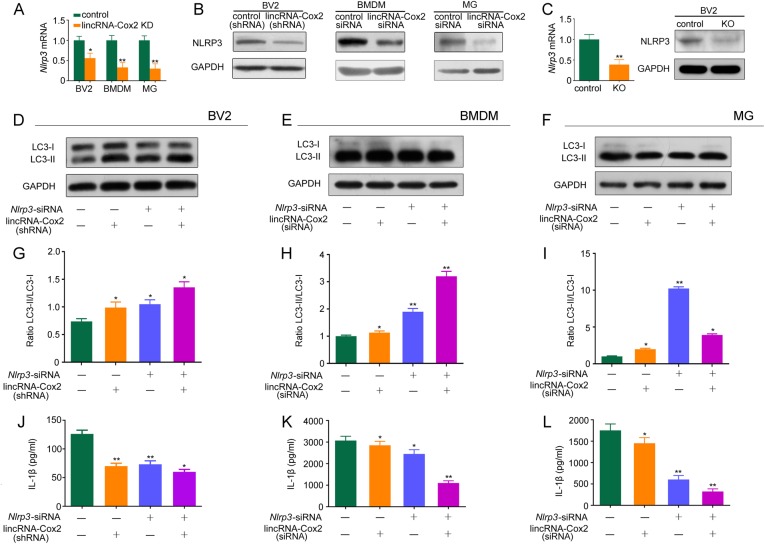
LincRNA-Cox2 knockdown upregulates autophagy by inhibiting Nlrp3 expression. **a**, **b** Q-PCR and Western blot detection of NLRP3 in control or lincRNA-Cox2 knockdown of BV2, BMDM and primary microglia treated with LPS for 4 h. **c** Q-PCR and Western blot detection of NLRP3 in control or lincRNA-Cox2 knockout (KO) of BV2. **d**–**f** Western blot of LC3-I and LC3-II in control or lincRNA-Cox2 knockdown of BV2, BMDM and primary microglia following treatment with *Nlrp3* siRNA and 4 h LPS add 1 mM ATP for 30 min administration. **g**–**i** Ratio of LC3-II/LC3-I in three independent experiments as in (**d**–**f**). **j**–**l** Levels of IL-1β secreted in control or lincRNA-Cox2 knockdown of BV2, BMDM and primary microglia following treatment with *Nlrp3* siRNA, 4 h LPS add 1 mM ATP for 30 min administration. GAPDH is shown as the loading control in all western blot figures (western blot repeated in three independent experiments). Columns are the mean values of triplicates; the error bar indicates the SEM. Asterisks indicate statistically significant difference from each other; **p < 0.01, *p < 0.05

Studies have shown that the NLRP3 inflammasome is activated by pattern associated molecular patterns (PAMPs) including LPS and danger/stress signals (ATP, monosodium urate crystals, and nigericin) [23–26]. The NLRP3 inflammasome is a caspase-1-activating multiprotein complex containing NLRP3, ASC and Caspase-1. The initial induction of autophagy in response to stimuli that trigger NLRP3 inflammasome activation was dependent on the inflammasome sensor but did not require complete assembly of the inflammasomes or IL-1β production [27]. We found that lincRNA-Cox2 knockdown decreased mRNA expression and translation of *Nlrp3* in BV2, BMDM and primary microglia (Fig. 2a, b). Furthermore, we deleted the lincRNA-Cox2 locus using CRISPR/Cas9 and confirmed that knockout of lincRNA-Cox2 decreased the mRNA expression and translation of *Nlrp3* in BV2 (Fig. 2c). We also found that *Nlrp3* downregulation in control group and lincRNA-Cox2 knockdown group resulted in an increase in the conversion of LC3-I to LC3-II in BV2, BMDM and primary microglia (Fig. 2d–f). The quantified results also showed that *Nlrp3* downregulation induced a marked increase in LC3-II/LC3-I ratio in BV2, BMDM and primary microglia (Fig. 2g–i). As the NLRP3 inflammasome is involved in caspase-1-dependent maturation of IL-1β in many contexts [28], we found that lincRNA-Cox2 knockdown weakened IL-1β production, and simultaneously the downregulation of *Nlrp3* by siRNA weakened IL-1β production in the control group and lincRNA-Cox2 knockdown group in BV2, BMDM, and primary microglia (Fig. 2j–l).

**Fig. 3:**
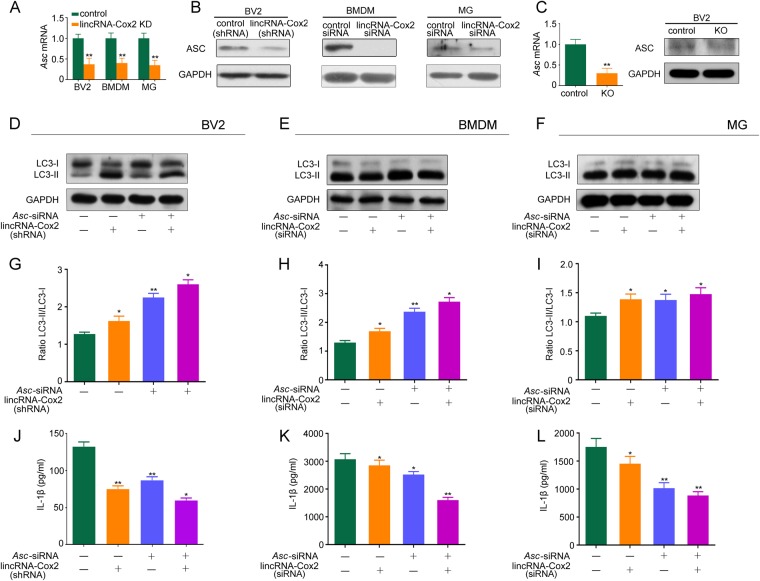
LincRNA-Cox2 knockdown upregulates autophagy by inhibiting Asc expression. **a**, **b** Q-PCR and Western blot detection of ASC in control or lincRNA-Cox2 knockdown of BV2, BMDM and primary microglia treated with LPS for 4 h. **c** Q-PCR and Western blot detection of ASC in control or lincRNA-Cox2 knockout(KO) of BV2. **d**–**f** Western blot of LC3-I and LC3-II in BV2, BMDM and primary microglia transfected with control siRNA or siRNA specific for *Asc*. **g**–**i** Ratio of LC3-II/LC3-I in three independent experiments as in **d**–**f**. **j**–**l** Levels of IL-1β secreted in control or lincRNA-Cox2 knockdown of BV2, BMDM and primary microglia following treatment with *Asc* siRNA, 4 h LPS and 30 min ATP (1 mM) administration. GAPDH is shown as the loading control in all western blot figures (western blot repeated in three independent experiments). Columns are the mean values; the error bar indicates the SEM. Asterisks indicate statistically significant differences from each other; **p < 0.01, *p < 0.05

We further found that lincRNA-Cox2 knockdown decreased mRNA expression and translation of the inflammasome adaptor *Asc* in BV2, BMDM and primary microglia (Fig. 3a, b). We further deleted the lincRNA-Cox2 locus using CRISPR/Cas9 to confirm that the knockout of lincRNA-Cox2 decreased the mRNA expression and translation of *Asc* in BV2 (Fig. 3c). Downregulation of Asc in control group and lincRNA-Cox2 knockdown group also increased the conversion of LC3-I to LC3-II in BV2, BMDM and primary microglia (Fig. 3d–f). The LC3II/LC3I ratio showed the same results (Fig. 3g–i). We also found *Asc* downregulation by siRNA in the control group and lincRNA-Cox2 knockdown group weakened IL-1β production in BV2, BMDM and primary microglia (Fig. 3j–l).

### LincRNA-Cox2 knockdown upregulates autophagy by inhibiting inflammasome activation

**Fig. 4:**
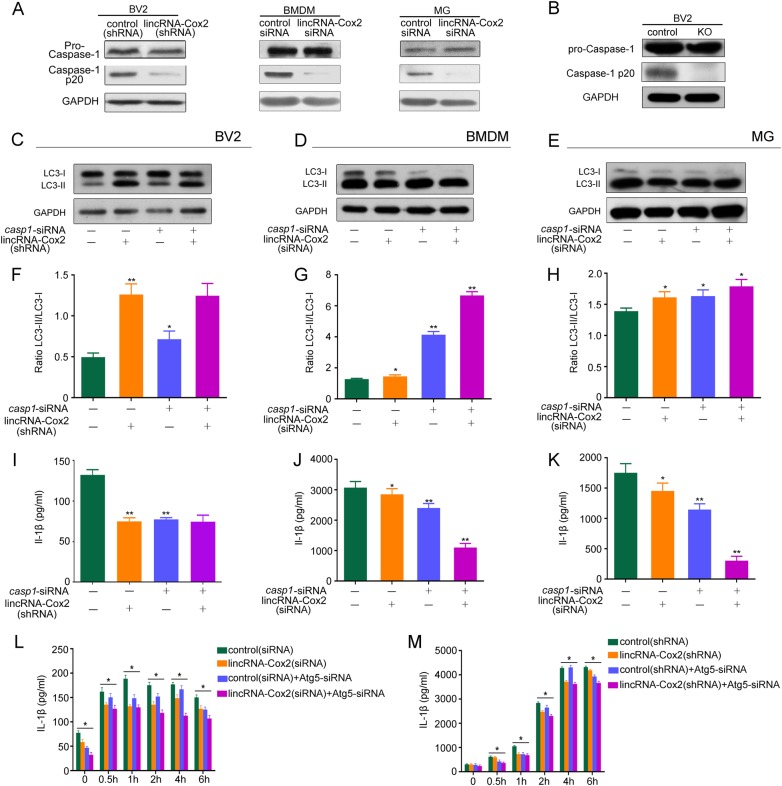
LincRNA-Cox2 knockdown upregulates autophagy by inhibiting caspase-1 activation. **a** Western blot of pro-Caspase-1 and Caspase-1 p20 in control or lincRNA-Cox2 knockdown of BV2, BMDM and primary microglia treated with LPS for 4 h and 30 min ATP (1 mM). **b** Western blot detection of pro-Caspase-1 and Caspase-1 p20 in control or lincRNA-Cox2 knockout (KO) of BV2. **c**–**e** Western blot of LC3-I and LC3-II in BV2, BMDM and primary microglia following treatment with *casp1* siRNA and 4 h LPS add 30 min ATP (1 mM) administration. **f**–**h** Ratio of LC3-II/LC3-I in three independent experiments as in **c**–**e**. **i**–**k** Levels of IL-1β secreted in control or lincRNA-Cox2 knockdown of BV2, BMDM and primary microglia following treatment with *casp1* siRNA, 4 h LPS and 30 min ATP (1 mM) administration. **l**, **m** Levels of IL-1β secreted in control or lincRNA-Cox2 knockdown of BV2 and BMDM treatment with *Atg5* siRNA, following LPS for different times and ATP (1 mM) 30 min administration. GAPDH is shown as the loading control in all western blot figures (western blot repeated in three independent experiments). Columns are the mean values; the error bar indicates the SEM. Asterisks indicate statistically significant differences from each other; **p < 0.01

The activated inflammasome cleaved pro-caspase-1 into mature caspase-1 (p20 and p10) and influenced the downstream target genes. Inflammasome-induced activation of caspase-1 down-regulates autophagy [29]. In this study, we found that downregulation of lincRNA-Cox2 decreased *Nlrp3* and *Asc* expression but did not have an effect on pro-caspase-1 levels in BV2, BMDM, and primary microglia (Fig. 4a). Further, we found that lincRNA-Cox2 knockdown inhibited caspase-1 activation in BV2, BMDM and primary microglia (Fig. 4a). We deleted the lincRNA-Cox2 locus using CRISPR/Cas9 to confirmed that the knockout of lincRNA-Cox2 did not influenced pro-caspase-1 levels but inhibited caspase-1 activation in BV2 (Fig. 4b). Knockdown of *Casp1* by siRNA in the control group and lincRNA-Cox2 knockdown group increased autophagy in BV2, BMDM and primary microglia (Fig. 4c–e). The LC3II/LC3I ratio showed the same results (Fig. 4f–h).We also found *Casp1* downregulation by siRNA in the control group and lincRNA-Cox2 knockdown group weakened IL-1β production in BV2, BMDM and primary microglia (Fig. 4i–k). To confirm the decreases in IL-1β secretion as a consequence of the effects of reduced inflammasome protein expression or what is a secondary consequence of autophagy-induced inflammasome suppression, we treated BV2 and BMDM by Atg5-siRNA in parallel with lincRNA-Cox2 knockdown with LPS for different times. The results showed the IL-1β secretion were decreased in lincRNA-Cox2 knockdown group at different times. But treated with Atg5-siRNA in parallel with lincRNA-Cox2 knockdown group did not showed increased IL-1β secretion (Fig. 4l, m). We also improved the lincRNA-Cox2 level in control and lincRNA-Cox2 knockdown group by lentivirus overexpressing of lincRNA-Cox2. The western blot results showed that NLRP3, ASC and Caspase-1 activation were increased in overexpressed groups, which protein level of the lincRNA-Cox2 knockdown group did not change as compared with the control group in the case of overexpression of lincRNA-Cox2, the ratio of LC3-II/LC3-I were also like this (Supplementary Fig. 3). Thus, inhibition of inflammasome activation and IL-1β secretion promoted autophagy following LPS stimulation, which could be mediated by lincRNA-Cox2 knockdown.

### LincRNA-Cox2 knockdown upregulates autophagy by inhibiting TRIF cleavage

**Fig. 5:**
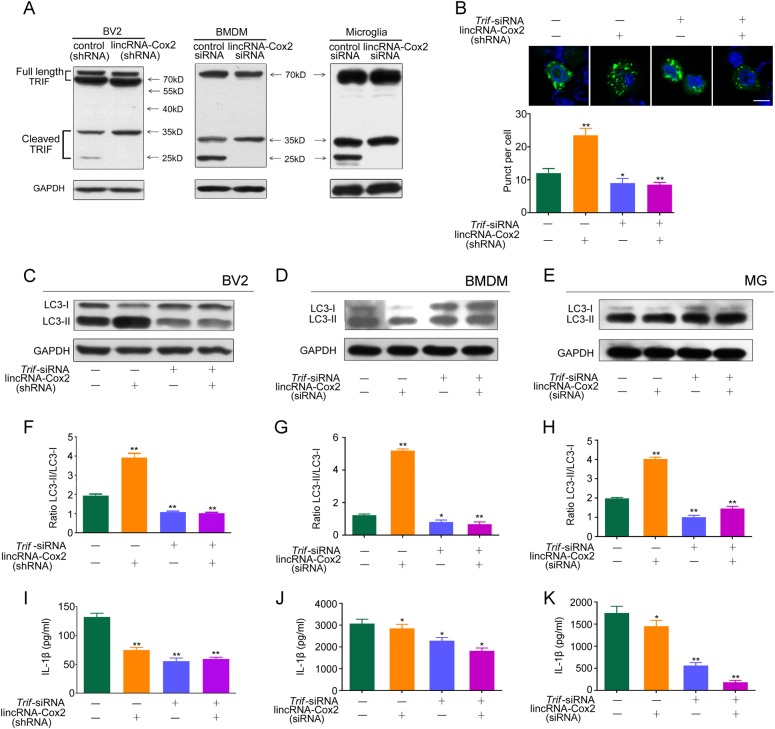
Upregulation of autophagy following lincRNA-Cox2 knockdown is mediated via TRIF. **a** Western blot of TRIF in control or lincRNA-Cox2 knockdown of BV2, BMDM and primary microglia treated with LPS for 4 h and 1 mM ATP for 30 min. **b** Representative confocal images of LC3 in control or lincRNA-Cox2 knockdown BV2 cells following treatment with *Trif* siRNA and 4 h LPS add 1 mM ATP for 30 min administration. LC3 staining is shown in green, and nuclei are blue. Scale bar indicates 10 μm (thee independent experiments). Numbers of LC3 puncta were counted. **c**–**e** Western blot of LC3-I and LC3-II in control or lincRNA-Cox2 knockdown of BV2, BMDM and primary microglia following treatment with *Trif* siRNA and 4 h LPS add 1 mM ATP for 30 min administration. **f**–**h** Ratio of LC3-II/LC3-I in three independent experiments as in **c**–**e**. **i**, **j**, **l** Levels of IL-1β secreted in control or lincRNA-Cox2 knockdown of BV2, BMDM and primary microglia following treatment with *Trif* siRNA, 4 h LPS and 1 mM ATP for 30 min administration. GAPDH is shown as the loading control in all western blot figures (western blot repeated in three independent experiments). Columns are the mean values of triplicates; the error bar indicates the SEM. Asterisks indicate statistically significant differences from each other; **p < 0.01

It has been reported that caspase-1 activation downregulated autophagy through TRIF cleavage [29]. So, we determined the role of TRIF in lincRNA-Cox2-regulated autophagy and found that lincRNA-Cox2 knockdown decreased TRIF cleavage compared with that in the control (Fig. 5a). The number of GFP-LC3 puncta was significantly decreased following TRIF knockdown (Fig. 5b). The conversion of LC3-I to LC3-II was significantly down-regulated in TRIF and lincRNA-Cox2 knockdown compared to that with lincRNA-Cox2 knockdown alone in BV2, BMDM and primary microglia (Fig. 5c–e). The LC3II/LC3I ratio showed the same results (Fig. 5f–h). TRIF knockdown by siRNA also inhibited IL-1β secretion in the control group and lincRNA-Cox2 knockdown group in BV2, BMDM and primary microglia (Fig. 5i–k). Thus, TRIF is an essential intermediate in lincRNA-Cox2-regulated autophagy following LPS stimulation, and the prevention of TRIF cleavage by caspase-1 led to the increased autophagy.

### LincRNA-Cox2-regulated of p65 nuclear translocation are recruited to the promoter region of *Nlrp3* and *Asc* genes following LPS stimulation

**Fig. 6:**
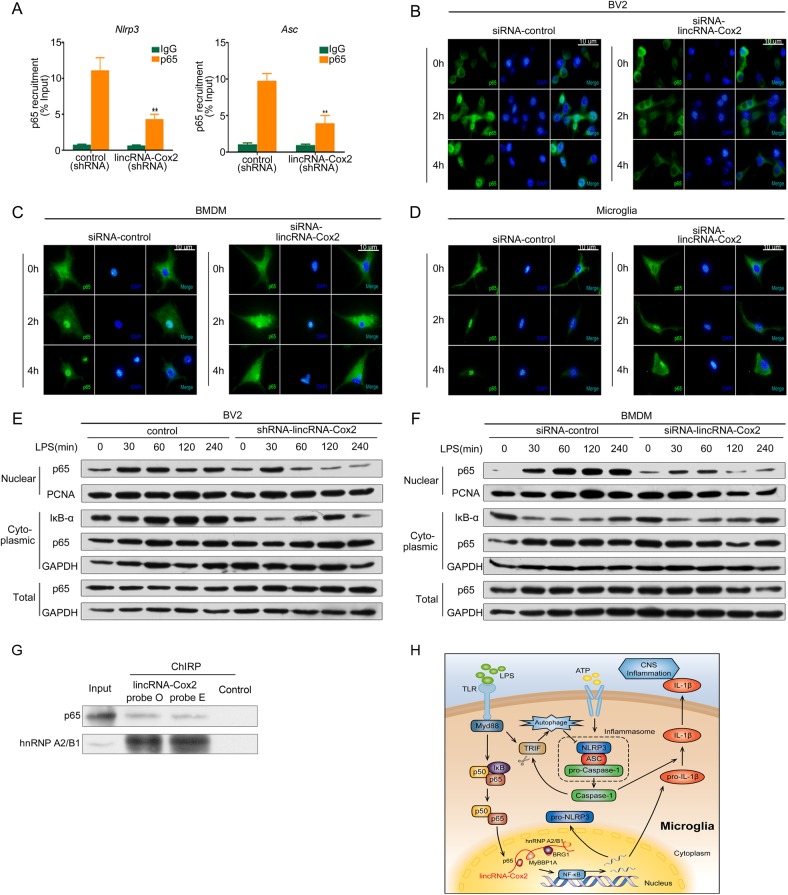
LincRNA-Cox2 knockdown inhibited p65 nuclear translocation to decrease inflammasome activation and enhance autophagy. **a** ChIP analysis of p65 recruitment to *Nlrp3* and *Asc* promoter region in BV2 cell line in response to LPS stimulation using anti-p65. **b**–**d** Intracellular localization of the p65 subunit of NF-κB was detected by immunofluorescence in BV2 cell line, BMDM and primary microglia cells following LPS stimulation for the indicated times. **e**, **f** Whole cell lysates were blotted using the p-65 antibody with GAPDH as loading control in BV2 and BMDM. Cytoplasmic extracts were blotted using the p-65 and IκB-α antibody with GAPDH as loading control in BV2 and BMDM. Nuclear extracts were blotted using the p-65 antibody with PCNA as loading control in BV2 and BMDM. **g** Proteins from lincRNA-Cox2 and control probe samples were analyzed by immunoblotting. **h** The mechanistic model for lincRNA-Cox2-mediated inflammasome activation and autophagy that regulates CNS inflammation

ChIP was used to evaluate the recruitment of p65 to the *Nlrp3* and *Asc* gene promoter region in BV2 cells following LPS simulation for 4 h. The results showed a significant decrease in p65 recruitment to the *Nlrp3* and *Asc* gene promoter region in lincRNA-Cox2 knockdown group (Fig. 6a). Next, we evaluated the nuclear translocation of p65 in BV2 cell line, BMDM and primary microglia cell following LPS stimulation for the indicated times by using immunofluorescence and Western blotting. The immunofluorescence showed the timing of p65 nuclear translocation in BV2, BMDM and primary microglia cell. The results showed that a significant decrease of p65 nuclear translocation in lincRNA-Cox2 knockdown group, especially for LPS stimulation for 4 h (Fig. 6b, c, d). The Western blotting results also showed that a significant decrease of p65 nuclear translocation in lincRNA-Cox2 knockdown group, especially for LPS stimulation for 4 h in BV2 and BMDM (Fig. 6e, f). We further employed chromatin isolation by RNA purification (ChIRP) followed by western blotting to analyze whether lincRNA-Cox2 directly binds key protein interactors, such as NF-κB p65 and hnRNP-A2/B1. The results showed that lincRNA-Cox2 can directly interact with NF-κB p65 and hnRNP-A2/B1 to assemble a complex that regulates gene function (Fig. 6g).

### LincRNA-Cox2 knockdown attenuates CNS inflammation and EAE severity and induces autophagy in microglia

**Fig. 7:**
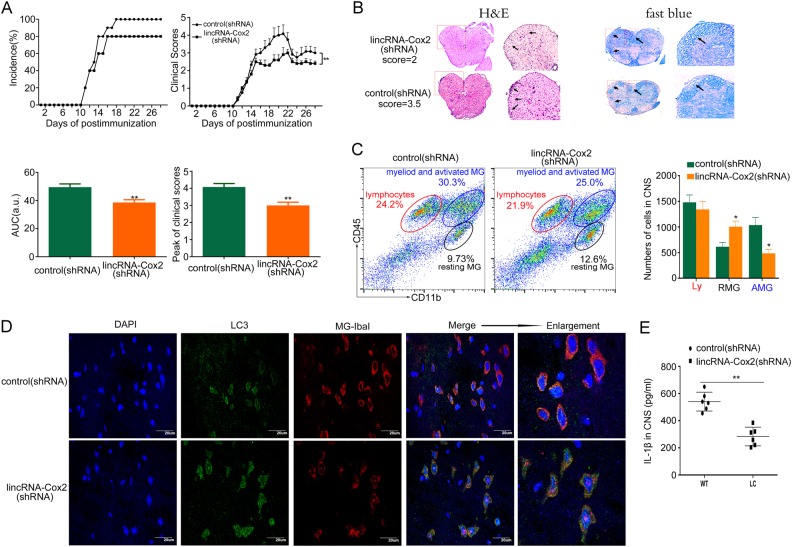
LincRNA-Cox2 knockdown attenuates CNS inflammation and EAE severity while inducing autophagy of microglia. **a** The incidence of disease, mean clinical score, area under the curve (AUC, arbitrary units) and peak of EAE mice were observed from day 1 to day 28 post-immunization for mice treated with control or lincRNA-Cox2 shRNA lentivirus by i.v. injection (*n* = 12 mice per group). The experiments were performed in triplicate on three separate occasions. One-way analysis of variance (ANOVA), where applicable, was performed to determine whether an overall statistically significant change existed between any two groups. The values represent the mean ± SD. **p < 0.01. **b** Hematoxylin and eosin (H&E)-stained sections show the infiltration of inflammatory cells into white matter. The arrows indicate inflammatory cells. Luxol fast blue staining shows the areas of intact myelin (blue) and demyelination (pink). The arrows indicate demyelination in the spinal cord, which includes sites associated with the infiltration of inflammatory cells. The mice were killed on Day 18 (the maximum difference) after immunization. **c** Flow cytometry analysis the percentage and cell numbers of immune cell infiltration into the CNS (brain and spinal cord) of MOG_35–55_-immunized control and lincRNA-Cox2 knockdown mice (*n* = 8 mice per group) on day 15 after immunization. The data are presented as a representative plot. **d** GFP-LC3 and Iba-I double-fluorescence colocalization analysis of microglia cell autophagy in the CNS. Blue is the cell nucleus, green labels autophagosomes, and red indicates microglia cells. **e** Levels of IL-1β secreted by CNS cells treated by LPS for 4 h in control or lincRNA-Cox2 knockdown mice (*n* = 6 mice per group) on day 15 after immunization. The error bar indicates the SEM. Asterisks indicate statistically significant differences from each other; *p < 0.05, **p < 0.01

EAE is an animal model of multiple sclerosis. EAE is induced by immunization with a CNS autoantigen and causes T cell–mediated inflammation and demyelination. The pathogenesis of EAE and multiple sclerosis is also mediated by microglia [30–32], which function in early embryonic development to generate the innate immune system in the CNS. Recent studies indicated that EAE development requires NLRP3 [33]. We next investigated the effects of lincRNA-Cox2 on CNS inflammation and autophagy *in vivo* in a mouse EAE model, and lincRNA-Cox2 knockdown reduced EAE severity (Fig. 7a). Disease severity was assessed by the area under the curve (AUC) and peak of clinical scores and was significantly lower in the lincRNA-Cox2 knockdown mice (Fig. 7a). We confirmed the efficiencies of lentivirus entering into the CNS and causing sufficient knockdown by GFP Immunofluorescence and qPCR(Supplementary Fig. 4). These data suggested that lincRNA-Cox2 knockdown significantly improved the clinical outcome of EAE. At the peak of the acute phase of the disease, H&E staining demonstrated that the lumbar spinal cords from lincRNA-Cox2 knockdown mice exhibited fewer inflammatory cells in the white matter (Fig. 7b). Sections stained with Luxol fast blue revealed that demyelination was markedly attenuated in the lincRNA-Cox2 knockdown mice (Fig. 7b). Furthermore, the Fig. 7c showed that the percentage and cell numbers of myeloid cells and activated resident microglia cells (CD11b^+^CD45^hi^) and resting microglia cells (CD11b^+^CD45^med^) changed obviously in CNS-infiltrating cells; the total lymphocytes (CD11b^−^CD45^hi^) changes were not significant. The resting microglia (CD11b^+^CD45^med^) were notable increased in the lincRNA-Cox2 knockdown group, CD11b^+^CD45^hi^ cells were reduced maybe partly due to the reduced proportion of activated resting microglia. So our studies mainly focused on the microglia of CNS. Next, we examined microglia autophagy by immunofluorescence and observed that the number of microglia performing autophagy was increased in the lincRNA-Cox2 knockdown group compared to that in the control group (Fig. 7d). The increase in autophagy might cause microglia quiescence and reduce IL-1β secretion.

## Discussion

LncRNAs serve important functions in numerous biological processes, including chromatin modification [34], RNA processing [35], structural scaffolds [36], modulation of apoptosis and invasion [37]. But how individual lncRNAs perform their activities involved in the activation of NLRP3 inflammasome and autophagy and contribute to neuroinflammation-dependent diseases remains poorly understood. LincRNA-Cox2 is localized to both the cytosolic and nuclear compartments and influences the expression of hundreds of inflammatory genes [18]. We found that lincRNA-Cox2 promote NF-κB p65 nuclear translocation and transcription, mediating *Nlrp3* and *Asc* expression and inflammasome activation. In previous studies, lincRNA-Cox2 is reported to be assembled into the SWI/SNF complex in cells after LPS stimulation [38]. Carpenter et al. [18] observed that lincRNA-Cox2 interacts with the heterogeneous nuclear ribonucleoprotein A/B and A2/B1 to activate and suppress immune response genes. However, SWI/SNF complex and heterogeneous nuclear ribonucleoprotein A/B and A2/B1 are all nuclear localization of proteins which may not regulated the NF-κB p65 nuclear translocation from cytoplasm to nucleus. We found that lincRNA-Cox2 directly binds NF-κB p65 to promote p65 transfer from cytoplasm to nucleus, which suggested that lincRNA-Cox2 and NF-κB might be coupled with other proteins and assembled into a complex in cytoplasm. When lincRNA-Cox2 was knocked down, the complex was resolved and reduced the relA/p65 translocation from cytoplasm to nucleus. Reduced p65 nuclear translocation and its binding to the *Nlrp3* and *Asc* promoter leaded to the decreased gene expression, which meanwhile explained the lincRNA-Cox2 knockdown-mediated extensive alteration in the expression profiles of inflammatory genes after TLR stimulation.

Autophagy was reported to be able to downregulate the activation of NLRP3 inflammasomes by removing damaged mitochondria [39]. Moreover, autophagy induction was dependent on inflammasome sensor NLRP3 but not on ASC or Caspase-1 [27]. Caspase-1-mediated TRIF cleavage is a key event controlling autophagy, type I interferon production and inflammasome activation with important functional consequences [29]. Caspase-1-processed cytokines IL-1β promotes the neuroinflammation [40]. We found that lincRNA-Cox2 knockdown inhibits NLRP3 inflammasome activation by downregulating NLRP3 and ASC mRNA and protein levels, which decreased caspase-1 activation and prevented cleavage of TRIF, thus enhancing autophagy. LincRNA-Cox2 knockdown-mediated weakened NLRP3 inflammasome activation resulted in limited pro-inflammatory IL-1β secretion by macrophages and microglia in vitro (Fig. 6h). Our results extended the understanding of lncRNA function to the crosstalk between NLRP3 inflammasome and autophagy, as well as their mediated neuroinflammation.

NLRP3 inflammasome activation causes a spectrum of neuroinflammation-dependent diseases such as EAE [41], which is a mouse model of the human disease multiple sclerosis (MS) that is characterized by CNS autoimmune inflammation associated with the activation of resident microglia and infiltration of encephalitogenic T cells and leukocytes from the periphery [42]. Several studies have shown that autophagy directly participates in the progression of MS or EAE [43–46]. However, whether and how individual lncRNAs target microglia and modulate EAE development remains largely unknown. Our results showed that myeloid cells and activated resident microglia cells (CD11b^+^CD45^hi^) and resting microglia cells (CD11b^+^CD45^med^) changed significantly in CNS-infiltrating cells from the lincRNA-Cox2-knockdown mice with EAE; the total lymphocytes (CD11b^−^CD45^hi^) changes were not significant. CD11b^+^CD45^hi^ cells were reduced maybe partly due to the reduced proportion of activated resting microglia in lincRNA-Cox2 knockdown. Moreover, lincRNA-Cox2 knockdown exhibited enhanced autophagy in microglia in vivo, which might contribute to microglia quiescence. Microglial quiescence in the CNS contributed to prevent the CNS inflammation [47]. Furthermore, lincRNA-Cox2 knockdown inhibited the NLRP3 inflammasomes activation and IL-1β secretion, which attenuated the neuroinflammation. Hence, lincRNA-Cox2 modulated the EAE development mainly by targeting macrophage and microglia. Here we showed that lincRNA-Cox2 acted both as a key regulator of microglia quiescence in the CNS and as a new modulator of macrophage and microglia activation during EAE. Our study also provided new insights into the original mechanism and new opportunities for therapeutic intervention in neuroinflammation-dependent diseases.

## Materials and methods

### Animals

Female C57BL/6 mice aged 6–8 weeks were purchased from the Academy of Military Medical Science (Beijing, China). The animals were housed and fed in a specific pathogen-free animal facility at the Experimental Animal Center of Tianjin Medical University (Tianjin, China). The experiments were performed in accordance with the guidelines for animal care and were approved by the Animal Ethics Committee of Tianjin Medical University (Tianjin, China).

### Materials

LPS (*E. coli* 055:B5, L2880) and ATP were purchased from Sigma-Aldrich (St. Louis, USA). 3-Methyladenine (3-MA) and chloroquine diphosphate (CQ) were purchased from InvivoGen. The GFP-LC3 vector was purchased from Hanbio (Shanghai, China).

### Cell culture and stimulation

BV2 mouse microglia was obtained from the Cell Resource Center, Peking Union Medical College. Cells were cultured in DMEM supplemented with 10% FBS (HyClone, GE) and antibiotics (100 IU/ml penicillin and 100 μg/ml streptomycin). Cells were stimulated with LPS(1 μg/ml) for the indicated times. Bone marrow cells from wild-type mice were cultured in DMEM with 10% fetal bovine serum and with recombinant M-CSF (10 ng/ml) to generate BMDMs. Cells were stimulated with LPS(1 μg/ml) for the indicated times. Mixed glial cultures were prepared from cerebral cortices of 1-day-old C57BL/6 mice according to the method of Giulian and Baker (1986). After mechanical and chemical dissociation, cortical cells were seeded in DMEM with 10% FBS at a density of 2.5 × 10^5^ cells/ml cultured at 37 °C in humidified 5% CO_2_/95% air. Medium was replaced every 2–3 days and confluency was achieved after 10–12 days in vitro. Microglia cells were obtained by shaking the flasks overnight at 200 rpm in accord with the shaking method of Giulian and Baker (1986). Floating cells were pelleted and subcultured at 4 × 10^5^ cells/ml on mixed glial-conditioned medium. Cells were stimulated with LPS (1 μg/ml) for the indicated times. For the NLRP3 inflammasome activation, cells were stimulated with LPS (1 μg/ml) for the indicated times and 1 mM ATP for 30 min.

### Cytokines

Cytokines in cell culture supernatants were measured by ELISA kits: Murine IL-1β, MultiSciences (Lianke) Biotech (Cat No. EK201B2). For CNS IL-1β measurement, the brain and spinal cord cells were isolated with 30%/70% percoll and culture with LPS (1 μg/ml) for 4 h; collected supernatants were measured by ELISA kits.

### Lentiviral constructs and infection

For RNA interference, we used a validation shRNA [18], which was cloned into the shRNA expression plasmid miRZip^TM^ (System Biosciences). The shRNA sequence for lincRNA-Cox2 was Forward: 5′-GATCCAAGAGTAAGATTCTGAAGATCCTCGAGGATCTTCAGAATCTTACTCTTTTTTTG-3′; Reverse: 5′-AATTCAAAAAAAGAGTAAGATTCTGAAGATCCTCGAGGATCTTCAGAATCTTACTCTTG-3′. For lincRNA-Cox2 overexpression, we cloned the full-length of lincRNA-Cox2 to pCDH-CMV-MCS-EF1-Puro lentvirus (SBI, Mountain View, CA, USA). The primer for full-length of lincRNA-Cox2 were below: Forward: GATTCCCTCTGCGTTTGCCTCCA; Reverse: GTTGAGATTATAATATAATTACA. Nine micrograms of vector was transfected into HEK293T cells with the packaging vectors pSpax (12.34 μg Addgene plasmid 12260) and pMD2 (6.71 μg Addgene plasmid 12259) using PEI (Polyscience). After 48 h and 72 h, the culture medium was collected and centrifuged at 2000 × *g* for 5 min to remove cell debris sedimentation, and the supernatant was membrane filtered. The samples were subsequently placed in 40 ml ultracentrifugation tubes, 1/4 volume PEG 8000 was added to supernatant, and the media were incubated overnight at 4 °C. The next day, the samples were centrifuged at 4000 rpm/min for 30 min at 4 °C and resuspended for virus precipitation with ice-cold sterile PBS to collect LV-shRNA-lincRNA-Cox2-GFP-Puro virus. The pCDH-CMV-MCS-EF1-lincRNA-Cox2-Puro lentvirus and negative control viruses LV-shRNA-control-GFP-Puro were obtained similarly. The BV2 cell line was transduced and selected using puromycin (5 μg/ml). Mice were infected with freshly purified virus (titer ≥ 1 × 10^8^ IU/ml) by tail vein injection. For 7–8 weeks old mice, 200 μl of concentrated virus solution (containing polybrene 8 μg/ml) was injected into each mouse.

### LincRNA-Cox2-CRISPR/Cas9 cell line construction and validation

CRISPR/Cas9 lentiviral construction method reference Zhang Feng’s article [48]. To establish a lentiviral CRISPR-Cas9-mediated knockout system, designed sgRNA sequences were constructed into the LentiCRISPR V2 (addgene). We used 2 validation gRNAs targeting both 5′ and 3′ sequences flanking the lincRNA-Cox2 locus [49]. The sgRNAs sequences are as follows:

lincRNA-Cox2-dnstrm-g1 ATCATTAACCTGTTATCATA;

lincRNA-Cox2-dnstrm-g2 CTTCAATAGACATATCTTTA;

lincRNA-Cox2-upstrm-g1 TCTTTGATGCAAGGAACTAC;

lincRNA-Cox2-upstrm-g2 TTACACTGTTTATCGCTGGT.

The sgRNAs included a 5-bp overhang for the forward (CACCG) and the reverse (CAAA) oligos to enable cloning into the BsmBI (Thermo Scientific, Waltham, MA) site of the LentiCRISPR V2. Positive clones were selected and the plasmids were further verified by DNA sequencing (Genewiz, Suzhou, China). We isolated clone using limited dilution and verified loss of lincRNA-Cox2 expression by qPCR.

### Small interfering RNAs and transfection

For gene silencing, small interfering RNA (siRNA) duplexes targeting the mouse gene were synthesized by Integrated DNA Technologies. The siRNA duplexes for mouse lincRNA-Cox2 knock down using the following oligos: 5′-GCCCUAAUAAGUGGGUUGUUU-3′ (sense sequence). The siRNA duplexes for mouse *Atg5*, *Nlrp3*, *Asc*, *Casp1*, *Trif*, and a siRNA-control were purchased from RiboBio (Guangzhou, China). The cells were treated with siRNAs (final concentration, 25 nM) using Lipofectamine RNAiMAX (Invitrogen, Carlsbad, CA, USA) according to the manufacturer’s instructions and were harvested 24 h after siRNA treatment. Quantitative RT-PCR and western blot was used to determine the significant expression changes for each target gene.

### Quantitative real-time PCR

RNA was extracted using TRIzol reagent (Invitrogen, Carlsbad, USA) in accordance with the manufacturer’s instructions. After RNA purification, the samples were treated with DNase to remove the contaminating genomic DNA. Reverse transcription was performed using random hexamers and M-MLV reverse transcriptase (Promega, Madison, USA). All other reverse transcription reagents were supplied by Takara (Takara, Japan). The gene-specific primers were synthesized at Genewiz(Suzhou, China). For relative quantitative real-time PCR, SYBR Green mix (Takara, Japan) was used in accordance with the manufacturer’s instructions. The reactions were performed in triplicate on an ABI PRISM 7500 Fast Real-Time PCR System (Applied Biosystems Inc., Foster City, California, USA), and the generated products were analyzed using ABI 7500 software (Version 2.0.5). The primer pairs for *Nlrp3* are listed below: Forward: 5′-ATTACCCGCCCGAGAAAGG-3′; Reverse: 5′-TCGCAGCAAAGATCCACACAG-3′. The primer pairs for *Asc* are listed below: Forward: 5′-CTTGTCAGGGGATGAACTCAAAA-3′; Reverse: 5′-GCCATACGACTCCAGATAGTAGC-3′. The primer pairs for lincRNA-Cox2 are listed below: Forward: 5′-TCCTTTCCCCCTCAATTCTT-3′; Reverse: 5′-TTTTCCCAATCTGCTTTGGT-3′.

### Western blot analysis

The cells were lysed in buffer containing 10 mM Tris-buffer (pH 7.6), 1% Triton X-100, 1% phosphatase inhibitor cocktail and 1 mM PMSF. The lysates were boiled in sodium dodecyl sulfate (SDS) sample buffer and were subjected to SDS–PAGE. Cytoplasmic extracts and nuclear extracts were obtained using the NE-PER Nuclear and Cytoplasmic Extraction Reagents (Sigma, 78833). Rabbit monoclonal antibodies against GAPDH, NLRP3, and ASC were purchased from Santa Cruz Biotechnology (CA, USA) and were diluted 1:1000. The anti-ATG5 antibody, anti-LC-3 antibody, anti-Caspase-1 antibody, anti-PCNA, and anti-p65 antibody were purchased from Cell Signaling Technology (Beverly, MA, USA) and were diluted 1:1000. The anti-TRIF antibody was purchased from Abcam and was diluted 1:1000. Horseradish peroxidase- conjugated goat anti-rabbit immunoglobulin G (Cell Signaling Technology, MA, USA) was used as the secondary antibody. Immunoreactive bands were identified using the ECL Western Blotting Detection System (Millipore Corporation, Billerica, MA, USA).

### Induction and treatment of EAE

For EAE induction, C57BL/6 mice (aged 6–8 wk) were immunized (s.c.) with 150 mg of myelin oligodendrocyte glycoprotein (MOG residues 35–55). The peptide sequence was Met-Glu-Val-Gly-Trp-Tyr-Arg-Ser-Pro-Phe-Ser-Arg-Val-Val-His-Leu-Tyr-Arg-Asn-Gly-Lys, and the purity was >95% (CL. Bio-Scientific CO., LTD., Xi’an, China). Immunization was performed by mixing the MOG35–55 peptide with complete Freund’s adjuvant containing 5 mg/ml of heat-killed H37Ra, a *Mycobacterium tuberculosis* strain (Difco Laboratories). Pertussis toxin (400 ng) (List Biological Laboratories) in PBS and 50 mM NaCl was administered i.p. on the day of immunization and again 24 h later. To treat EAE, LV-shRNA-control and LV-shRNA-lincRNA-Cox2 were administered through intravenous injections 7 days before and after immunization. The mice were weighed and examined daily for disease symptoms, which were assessed using the following standard score system: 0, no obvious changes in motor functions; 1.0, limp tail; 2.0, limp tail and wobbly gait; 3.0, bilateral hind limb paralysis; 4.0, complete hind limb and partial fore limb paralysis; and 5.0, death.

### Histopathology and immunohistochemistry

The spinal cords from mice transcardially perfused with 4% paraformaldehyde were dissected and postfixed overnight. Paraffin-embedded 5–10 μm spinal cord sections were stained with hematoxylin-eosin (H&E) for routine histological analysis of inflammatory infiltration and with Luxol fast blue (Alfa Aesar, Ward Hill, USA) for evaluation of demyelination. For confocal imaging of microglia and autophagosome colocalization, the mouse anti-IbaI (Abcam) and anti-LC3II antibody (Cell Signaling Technology) were used for immunostaining. Donkey anti-rabbit and donkey anti-goat conjugated to Alexa Fluor 488 or Alexa Fluor 594 were used as secondary antibodies (all from Proteintech). The Olympus FluoView FV1000 Microscope equipped with a ×100 objective was used for cell imaging. For p65 immunofluorescence, cells were cultured on glass coverslips, mouse anti-p65 (1:400) was the primary antibody and Alexa Fluor 488-labeled goat anti-rabbit antibody (1:1000) was the secondary antibody. Coverslips were mounted and stored at 4 °C for further investigation.

### Confocal microscopy

For confocal images, stable lincRNA-Cox2 knockdown BV2 cells were seeded at 2 × 10^5^/ml the day before experiments on 35-mm glass-bottom culture dishes. The following day, the overnight medium was replaced with fresh medium and transfected with the siRNA using LipoRNAMax for 48 h. The cells were then stimulated with LPS for 4 h and 1 mM ATP for 30 min. The cells were fixed with 4% paraformaldehyde for 20 min, and then imaging was performed on an Olympus FluoView FV1000 microscope equipped with a ×100 objective. For quantification of LC3 puncta, image analysis was performed using ImageJ software (NIH, Maryland). All of the results show the mean number of puncta per cell; for each analysis at least 30 cells were analyzed.

### Chromatin immunoprecipitation (ChIP)

Chromatin immunoprecipitation (ChIP) analysis was performed with a commercially available ChIP Assay Kit (Affymetrix Chromatin Immunoprecipitation Assay), in accordance with the manufacturer’s instructions. In brief, the chromatin fraction was immunoprecipitated overnight at 4 °C using the Ab. PCR amplification was performed with specific primers. The percentage input method was used to normalize ChIP data. Signals obtained from the ChIP were divided by signals obtained from an input (1% of starting chromatin was used as input). All oligo sequences were designed using Primer3web (http://primer3.wi.mit.edu/), and were targeted within the 500 nucleotides region downstream of the transcription start site of each gene. The primer sequences used for detecting the p65 recruitment are below: *Nlrp3* (Forward: 5′-GGGTTCTGGTCAGACACGAG-3′ and Reverse: 5′-GTCCTGAGCCATGGAAGAAA-3′), *Asc* (Forward: 5′-GAAGCTGCTGACAGTGCAAC-3′ and Reverse: 5′-AGCTCCAAGCCATACGACTC-3′). The Abs were used for ChIP analysis from CST (anti-p65, 8242). Experiments were performed three times with independent chromatin samples and yielded similar results.

### Chromatin Isolation by RNA Purification (ChIRP)

Cell harvesting, lysis, disruption, and ChIRP were was performed, as previously described [50], followed some modification as described [51]. Briefly, the lincRNA-Cox2: chromatin complex was captured by a pool of tiling oligonucleotide probes specific to lincRNA-Cox2. The probes were labeled and separated into two pools; “odd” pool contains the probe 1, 3, 5 etc. and “even” pools contains the probe 2, 4, 6 etc. The chromatin complexes were confirmed by western blot. The control probe targets *LacZ* mRNA were used as which is not expressed in mammalian cells. Oligos were synthesized with 3′ biotin-TEG modification at Genewiz (Suzhou, China). The probe sequences were listed in Table 1.

**Table 1:** Sequences of ChIRP Probes for llincRNA-Cox2 used in this study, related to Fig. 7e

Probe Name	5′-3′ sequence
Ctrl probe	ccagtgaatccgtaatcatg
lincRNA-Cox2 probe 1	aggcaaacgcagagggaatc
lincRNA-Cox2 probe 2	aaagccggctttttcacaac
lincRNA-Cox2 probe 3	tgcatgttttcttttgtcga
lincRNA-Cox2 probe 4	ggctgagtgagttaacgaga
lincRNA-Cox2 probe 5	accacattaggaaagtggca
lincRNA-Cox2 probe 6	ttaagttgtcaacctccttt
lincRNA-Cox2 probe 7	ccacttttgcagtctaagtt
lincRNA-Cox2 probe 8	ctgctagccattatcctaaa
lincRNA-Cox2 probe 9	gaaaagtttccaggaaggga

### RNA FISH

RNA FISH were carried out as described previously [52]. Probes used for RNA FISH were lincRNA-Cox2 probe 1 (see Table 1). The probes were synthesized and labeled with 3′ biotin-TEG. Prior to hybridization with cells, the specific probe attached to the coverslips was denatured at 75 °C for 5 min. The denatured probe was dissolved in hybridization buffer (1 mg/ml of BSA, 2 × SSC, 20% dextran sulfate) and hybridized to the cells at 37 °C overnight. After three washes in 2 × SSC for 10 min, the slides were mounted with 90% (v/v) glycerol. Controls including an unrelated RNA probe were prepared and used according to the steps described above.

### Statistical analysis

The data are presented as average values ± SEM from multiple individual experiments each performed in triplicate or as average values ± SD from triplicate measurements in a representative experiment. Statistical analysis was performed using a nonparametric Mann–Whitney *U*-test, an unpaired two-tailed *t*-test. *p* < 0.05 was considered statistically significant.

### Significance statement

Inflammasome activation and autophagy are fundamental eukaryotic pathways that have multiple effects on a series of diseases including cancer, diabetes, atherosclerosis, and neurodegenerative diseases. Our work elucidates the link between lincRNA-Cox2 and the inflammasome-autophagy crosstalk in macrophage and microglia, revealing a role for lncRNAs in activation of NLRP3 inflammasome and autophagy. As a representative of neuroinflammation-dependent diseases, multiple sclerosis (MS) is believed to be caused by Th17 cells and microglia-mediated central nervous system (CNS) inflammation and demyelination. Experimental autoimmune encephalomyelitis (EAE) serves as an ideal animal model for MS. Here, we demonstrate that lincRNA-Cox2 knockdown results in protection from EAE, and exhibits notable increased resting microglia (CD11b^+^CD45^med^), inhibited the IL-1β secretion and enhanced autophagy in CNS, which might provide new insights into the original mechanism and new opportunities for therapeutic intervention in neuroinflammation-dependent diseases.

## Electronic supplementary material

Supplementary figure legends(DOC 31 kb)

Supplementary figure 1(TIF 2282 kb)

Supplementary figure 2(TIF 982 kb)

Supplementary figure 3(TIF 709 kb)

Supplementary figure 4(TIF 2081 kb)
