# Supplementary material for: lincRNA-Cox2 regulates NLRP3 inflammasome and autophagy mediated neuroinflammation
Source: Cell Death Differ. 2018 Apr 17;26(1):130–45. doi: 10.1038/s41418-018-0105-8 (PMC6294802; doi:10.1038/s41418-018-0105-8)
Supplement: Supplementary file 1 — Supplementary figure legends(DOC 31 kb) [file 41418_2018_105_MOESM1_ESM.doc]

**Supplementary Figure Legends**

**Figure S1. LincRNA-Cox2 expressions were downregulated in lincRNA-Cox2 knockdown cells.**

(A) Q-PCR detection of lincRNA-Cox2 expressions in control or lincRNA-Cox2 knockdown of BV2, BMDM and BV2 knockout of the lincRNA-Cox2 treated with LPS for different times. (B) RNA FISH detection of lincRNA-Cox2 expressions in control or lincRNA-Cox2 knockdown of BMDM treated with LPS for different times.

**Figure S2. LincRNA-Cox2 knockout upregulates the autophagy by inhibiting Nlrp3 inflammasome.**

(A) Western blot and Ratio of LC3-II and LC3-I in control or lincRNA-Cox2 knockout of BV2 following treatment with *Nlrp3* siRNA and 4 h LPS add 1 mM ATP for 30 min administration. (B) Western blot and Ratio of LC3-II and LC3-I in control or lincRNA-Cox2 knockout of BV2 following treatment with *Asc* siRNA and 4 h LPS add 1 mM ATP for 30 min administration. (C) Western blot and Ratio of LC3-II and LC3-I in control or lincRNA-Cox2 knockout of BV2 following treatment with *Casp1* siRNA and 4 h LPS add 1 mM ATP for 30 min administration.

**Figure S3. LincRNA-Cox2 regulates macrophage and microglia autophagy through NLRP3 inflammasome.**

(A) lincRNA-Cox2 overexpression promoted the NLRP3 inflammasome activation and inhibited the autophagy in BV2 cells. (B) lincRNA-Cox2 overexpression promoted the NLRP3 inflammasome activation and inhibited the autophagy in BMDM.

**Figure S4. The efficiencies of lentivirus caused knockdown in periphery and CNS.**

(A) Q-PCR detection of the efficiencies of lentivirus caused lincRNA-Cox2 knockdown in periphery and CNS. (B) GFP Immunofluorescence detection of the efficiencies of lentivirus entered into the microglia cells of CNS.
