# Supplementary figures and images for: lincRNA-Cox2 regulates NLRP3 inflammasome and autophagy mediated neuroinflammation
Source: Cell Death Differ. 2018 Apr 17;26(1):130–45. doi: 10.1038/s41418-018-0105-8 (PMC6294802; doi:10.1038/s41418-018-0105-8)

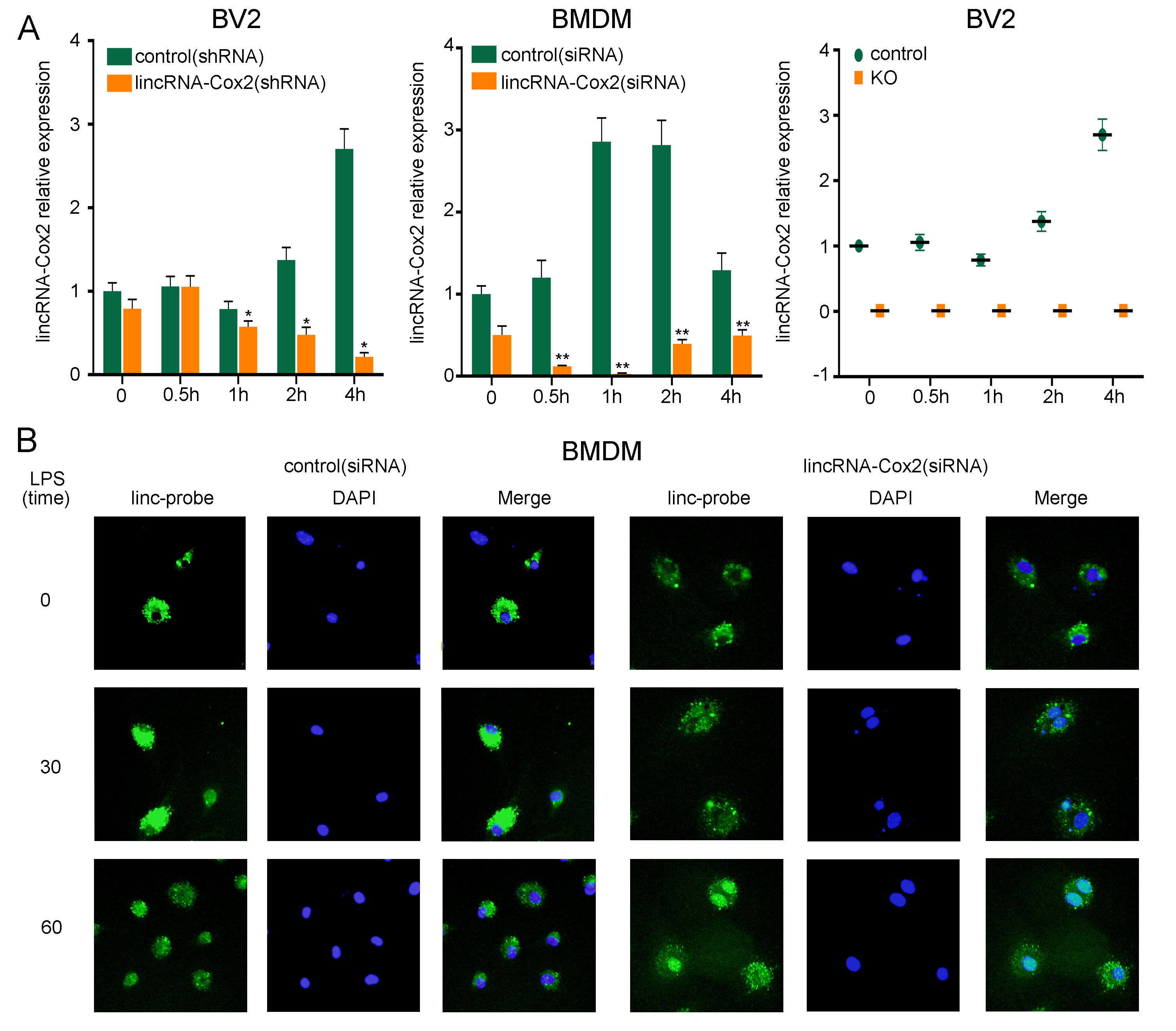

Supplement: Supplementary file 2 — Supplementary figure 1(TIF 2282 kb) [file 41418_2018_105_MOESM2_ESM.tif]

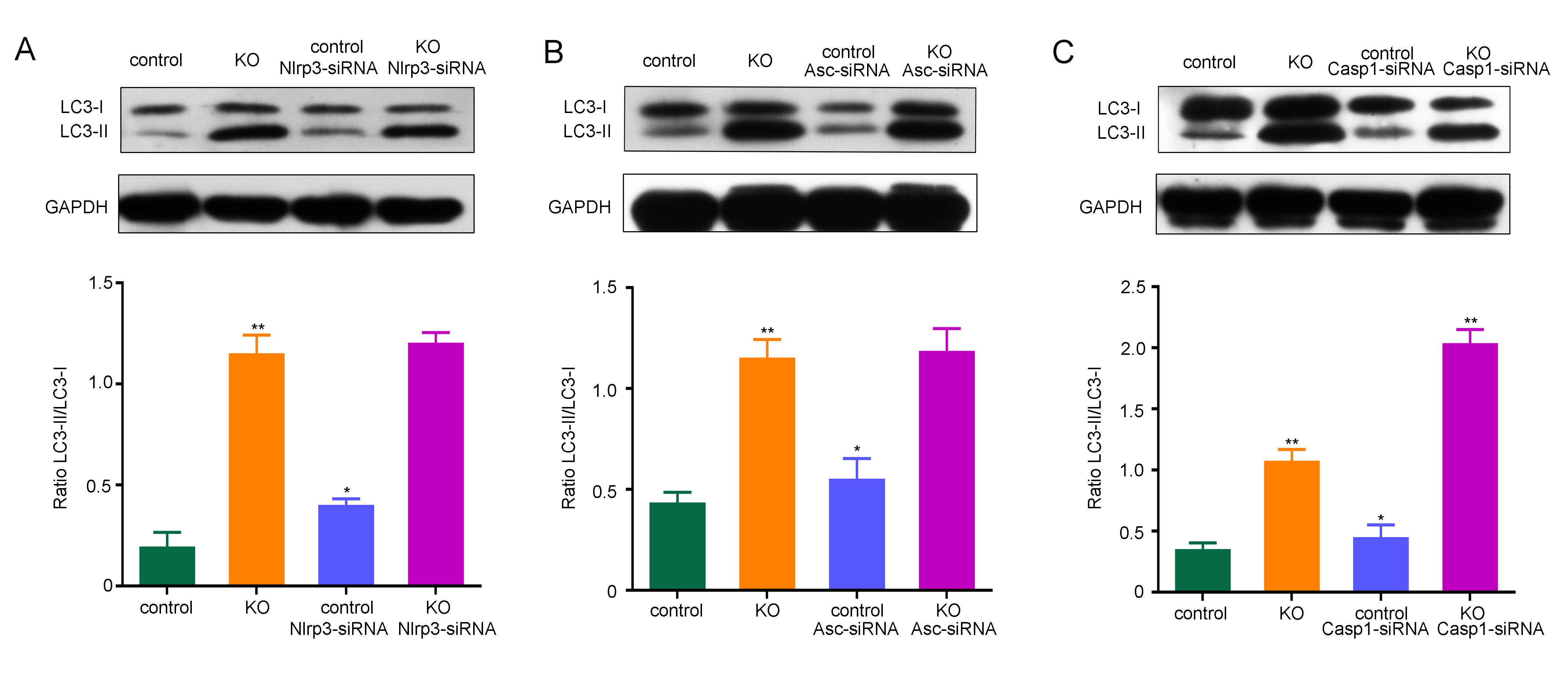

Supplement: Supplementary file 3 — Supplementary figure 2(TIF 982 kb) [file 41418_2018_105_MOESM3_ESM.tif]

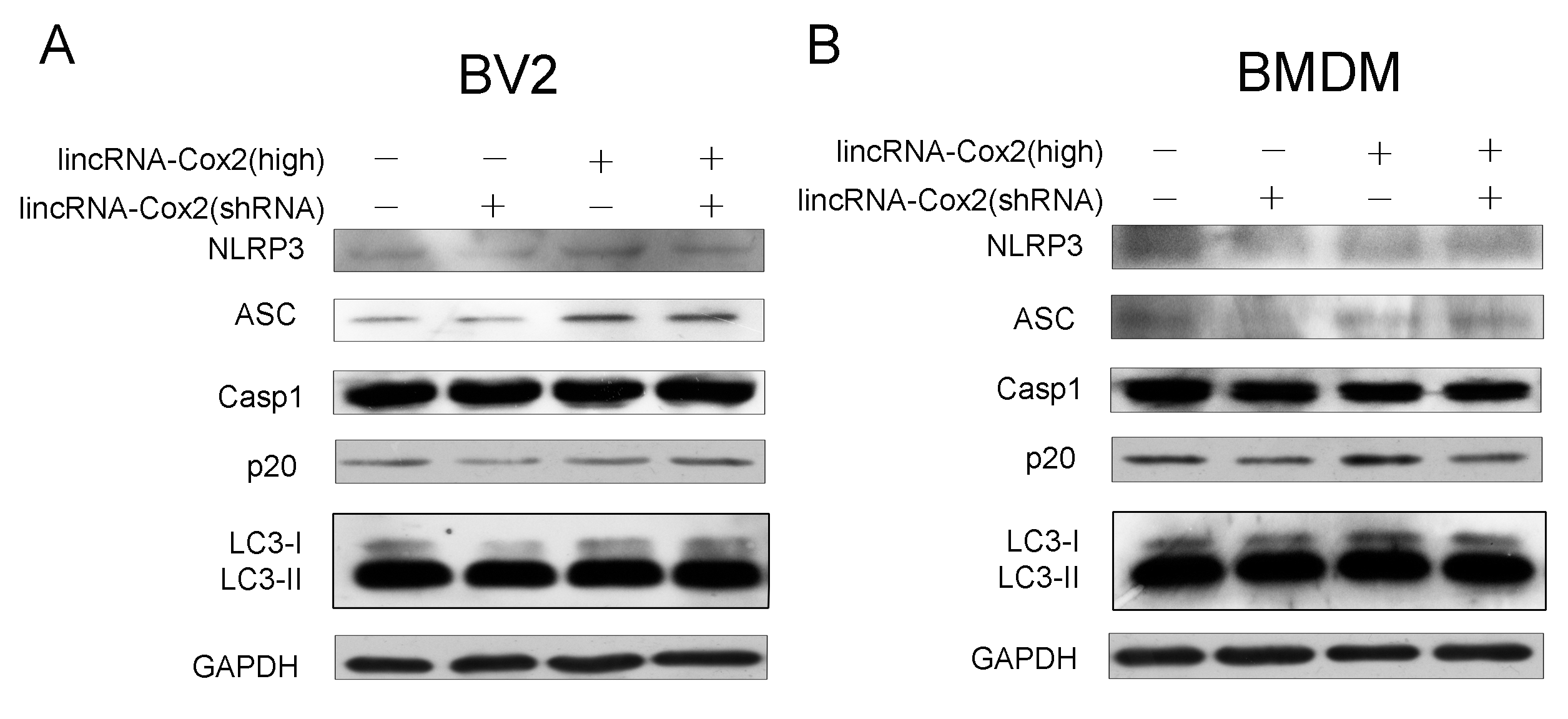

Supplement: Supplementary file 4 — Supplementary figure 3(TIF 709 kb) [file 41418_2018_105_MOESM4_ESM.tif]

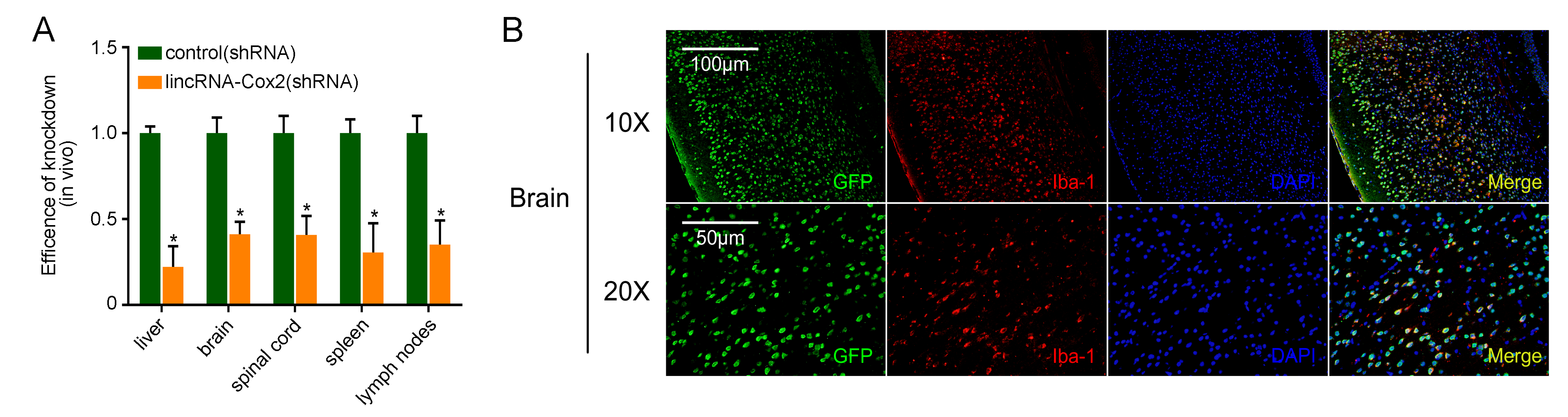

Supplement: Supplementary file 5 — Supplementary figure 4(TIF 2081 kb) [file 41418_2018_105_MOESM5_ESM.tif]
